# Fine-Tuning Bidirectional Encoder Representations From Transformers (BERT)–Based Models on Large-Scale Electronic Health Record Notes: An Empirical Study

**DOI:** 10.2196/14830

**Published:** 2019-09-12

**Authors:** Fei Li, Yonghao Jin, Weisong Liu, Bhanu Pratap Singh Rawat, Pengshan Cai, Hong Yu

**Affiliations:** 1 Department of Computer Science University of Massachusetts Lowell Lowell, MA United States; 2 Center for Healthcare Organization and Implementation Research Bedford Veterans Affairs Medical Center Bedford, MA United States; 3 Department of Medicine University of Massachusetts Medical School Worcester, MA United States; 4 School of Computer Science University of Massachusetts Amherst, MA United States

**Keywords:** natural language processing, entity normalization, deep learning, electronic health record note, BERT

## Abstract

**Background:**

The bidirectional encoder representations from transformers (BERT) model has achieved great success in many natural language processing (NLP) tasks, such as named entity recognition and question answering. However, little prior work has explored this model to be used for an important task in the biomedical and clinical domains, namely entity normalization.

**Objective:**

We aim to investigate the effectiveness of BERT-based models for biomedical or clinical entity normalization. In addition, our second objective is to investigate whether the domains of training data influence the performances of BERT-based models as well as the degree of influence.

**Methods:**

Our data was comprised of 1.5 million unlabeled electronic health record (EHR) notes. We first fine-tuned BioBERT on this large collection of unlabeled EHR notes. This generated our BERT-based model trained using 1.5 million electronic health record notes (EhrBERT). We then further fine-tuned EhrBERT, BioBERT, and BERT on three annotated corpora for biomedical and clinical entity normalization: the Medication, Indication, and Adverse Drug Events (MADE) 1.0 corpus, the National Center for Biotechnology Information (NCBI) disease corpus, and the Chemical-Disease Relations (CDR) corpus. We compared our models with two state-of-the-art normalization systems, namely MetaMap and disease name normalization (DNorm).

**Results:**

EhrBERT achieved 40.95% F1 in the MADE 1.0 corpus for mapping named entities to the Medical Dictionary for Regulatory Activities and the Systematized Nomenclature of Medicine—Clinical Terms (SNOMED-CT), which have about 380,000 terms. In this corpus, EhrBERT outperformed MetaMap by 2.36% in F1. For the NCBI disease corpus and CDR corpus, EhrBERT also outperformed DNorm by improving the F1 scores from 88.37% and 89.92% to 90.35% and 93.82%, respectively. Compared with BioBERT and BERT, EhrBERT outperformed them on the MADE 1.0 corpus and the CDR corpus.

**Conclusions:**

Our work shows that BERT-based models have achieved state-of-the-art performance for biomedical and clinical entity normalization. BERT-based models can be readily fine-tuned to normalize any kind of named entities.

## Introduction

### Background

Entity normalization (EN) is the process of mapping a named entity mention (eg, dyspnea on exertion) to a term (eg, 60845006: Dyspnea on exertion) in a controlled vocabulary (eg, Systematized Nomenclature of Medicine—Clinical Terms [SNOMED-CT]) [1]. It is a significant task for natural language processing (NLP) [2]. It is also an important step for other NLP tasks such as knowledge base construction and information extraction [3-6].

EN has been extensively studied in the biomedical and clinical domains [7,8]. Supervised approaches usually perform better than unsupervised approaches. However, their performance depends highly on the quantity and quality of annotated data [1,8-10]. Recently, deep representation-learning models, such as bidirectional encoder representations from transformers (BERT) and embeddings from language models (ELMo), have been shown to improve many NLP tasks [11,12]. These studies usually employ unsupervised pretraining techniques to learn language representations from large-scale raw text.

Deep representation-learning models learn word representations from large-scale unannotated data, which are more generalizable than the models trained only from annotated data with limited sizes. Therefore, deep representation-learning models can be fine-tuned to improve downstream NLP tasks. For example, BERT [11] has achieved new state-of-the-art results on 11 NLP tasks, including question answering and natural language inference. BioBERT [13], which has a similar architecture but was pretrained using PubMed and PubMed Central (PMC) publications, achieved new state-of-the-art results on three biomedical NLP tasks: named entity recognition, relation extraction, and question answering. However, little work has explored such models in biomedical and clinical entity normalization tasks.

### Related Work

Previous work has studied various language models. For instance, the n-gram language model [2] assumes that the current word can be predicted via previous *n* words. Bengio et al [14] utilized feed-forward neural networks to build a language model, but their approach was limited to a fixed-length context. Mikolov et al [15] employed recurrent neural networks to represent languages, which can theoretically utilize an arbitrary-length context.

Besides language models, researchers have also explored the problem of word representations. The bag-of-words model [16] assumes that a word can be represented by its neighbor words. Brown et al [17] proposed a clustering algorithm to group words into clusters that are semantically related. Their approach can be considered as a discrete version of distributed word representations. As deep learning develops, some researchers leveraged neural networks to generate word representations [16,18].

Recently, researchers have found that many downstream applications can benefit from the word representations generated by pretrained models [11,12]. ELMo utilized bidirectional recurrent neural networks to generate word representations [12]. Compared to word2vec [16], their word representations are contextualized and contain subword information. BERT [11] utilizes two pretraining objectives, *mask language model* and *next sentence prediction*, which can naturally benefit from large unlabeled data. The BERT input consists of three parts: word pieces, positions, and segments. BERT uses bidirectional transformers to generate word representations, which are jointly conditioned on both the left and right context in all layers. BERT and its derivatives such as BioBERT [13] achieved new state-of-the-art results on various NLP or biomedical NLP tasks (eg, question answering, named entity recognition, and relation extraction) through simple fine-tuning techniques.

In this paper, we investigated the effectiveness of such an approach in a new task, namely, biomedical or clinical entity normalization. In the biomedical or clinical domain, MetaMap [19] is the tool that is widely used to extract terms and link them to ﻿the Unified Medical Language System (UMLS) Metathesaurus [3]. Researchers utilized MetaMap in various scenarios, such as ﻿medical concept identification in electronic health record (EHR) notes [20], ﻿ vocabulary construction for consumer health [21], and ﻿text mining from patent data [22]. In this paper, we employed MetaMap as one of our baselines. Previous work consisting of entity normalization can be roughly divided into three types: (1) rule-based approaches [7] depend on manually designed rules, but they are not able to cover all situations; (2) similarity-based approaches [23] compute similarities between entity mentions and terms, but the metrics of similarities highly influence the performances of such approaches; (3) machine learning-based approaches [1,8-10] can perform better, but they usually require enough annotated data to train models from scratch. In this paper, we fine-tuned pretrained representation-learning models on the entity normalization task to show that they are more effective than traditional supervised approaches.

### Objective

**Figure 1:**
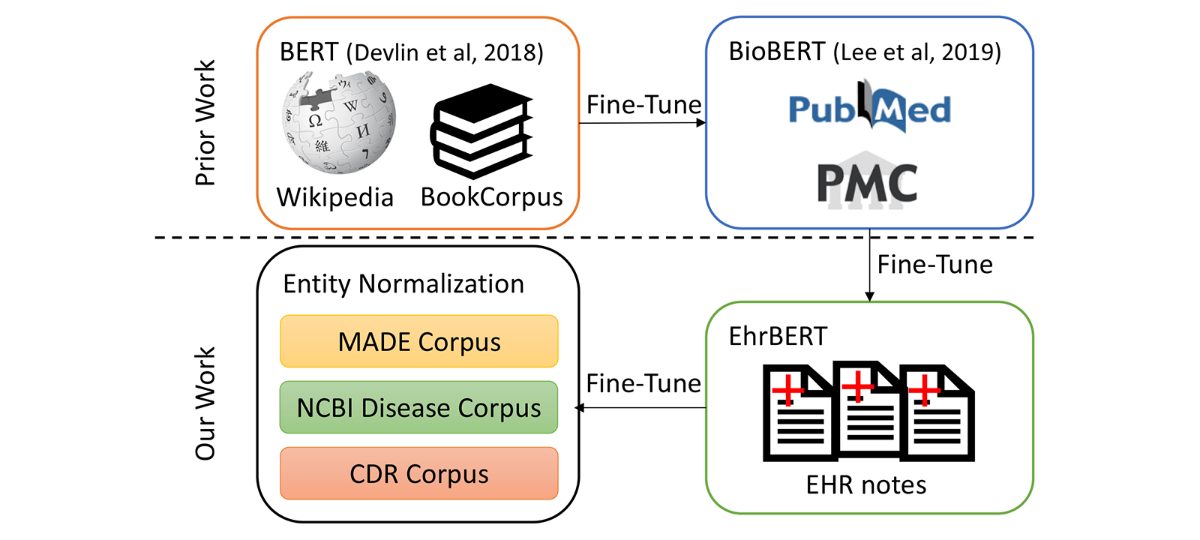
Overview of this paper's methods. Bidirectional encoder representations from transformers (BERT) [11] was trained on Wikipedia text and the BookCorpus dataset. BioBERT [13] was initialized with BERT and fine-tuned using PubMed and (PubMed Central) PMC publications. We initialized the BERT-based model that was trained using 1.5 million electronic health record notes (EhrBERT) with BioBERT and then fine-tuned it using unlabeled electronic health record (EHR) notes. We further fine-tuned EhrBERT using annotated corpora for the entity normalization task. CDR: Chemical-Disease Relations; MADE: Medication, Indication, and Adverse Drug Events; NCBI: National Center for Biotechnology Information.

In this study, we proposed the following objectives:

1. We aimed to explore the effectiveness of BERT-based models for the entity normalization task in the biomedical and clinical domains. The overview of this paper’s methods is shown in Figure 1.
2. We aimed to investigate whether the domains of training data influence the performances of BERT-based models as well as the degree of influence.

### Contributions

The main contributions of this paper are as follows:

1. We proposed a BERT-based model that was trained using 1.5 million EHR notes (EhrBERT). To facilitate the research of clinical NLP, the EhrBERT is publicly available at GitHub [24].
2. We evaluated EhrBERT on three entity normalization corpora in the biomedical and clinical domain. EhrBERT improved the F1s in three corpora by 2.36%, 1.98%, and 3.9% compared with state-of-the-art models such as MetaMap and disease name normalization (DNorm). EhrBERT also performed better than BioBERT and BERT in two corpora.
3. By comparing BERT, BioBERT, and EhrBERT, we found that the domain influences the performances of BERT-based models. However, if the domains of models and tasks are close, such an effect is generally not statistically significant. However, if their domains are distant, such an effect becomes large.

## Methods

### Overview

In this section, we will first describe how to generate the clinical representation-learning model using BERT and EHR notes. Next, the details of the models used for entity normalization will be introduced. Lastly, we will introduce the corpora used in this paper. Throughout this paper, we leveraged the PyTorch implementation of BERT developed by Hugging Face [25] to implement our models.

### A BERT-Based Model Trained on Electronic Health Record Notes

With the approval from the Institutional Review Boards at the University of Massachusetts Medical School, we collected approximately 1.5 million EHR notes from the UMass Memorial Medical Center. To investigate whether the data size influences the performance of EhrBERT, we split these EHR notes into a smaller part (500,000 notes) and a larger part (1 million notes). Throughout this paper, we will refer to them and their corresponding models as EhrBERT_500k_ and EhrBERT_1M_, respectively.

For preprocessing, EHR notes were first split into sentences. Since the format of EHR notes is special, we did not only employ the period and line break as sentence splitters, but also other symbols such as the tab. After sentence splitting, we utilized the Natural Language Toolkit [26] for tokenization. Regarding EhrBERT_500k_, the total token number is approximately 295 million and the sentence number is approximately 25 million. Therefore, the average sentence length is 11.6 tokens. Regarding EhrBERT_1M_, the total token number is approximately 598 million, the sentence number is approximately 55 million, and the average sentence length is approximately 10.8 tokens.

After data preparation, we applied BioBERT [13] as the starting point to train EhrBERT. Since BioBERT keeps the identical setting as BERT [11] but pretrains the model via PubMed and PMC data, its domain is much closer to ours. In addition, since BioBERT was initialized with BERT, our model can benefit from both BERT and BioBERT.

The main hyper-parameters used to train EhrBERT are listed in Table 1.

**Table 1:** Main hyper-parameter settings of EhrBERT^a^.

Hyper-parameter	Value
Epoch	15
Maximal sequence length	128
Batch size	64
Learning rate	0.00003
Embedding size	768
Dropout probability	0.1
Transformer blocks	12
Self-attention heads	12

^a^EhrBERT: bidirectional encoder representations from transformers (BERT)–based model that was trained using 1.5 million electronic health record notes.

We utilized 15 epochs to train EhrBERT, which were selected based on prior work [27] and our data size. Based on the average sentence length in our data, the maximal sequence length was set as 128, which is shorter than that used by BERT. The batch size and learning rate were set as 64 and 0.00003, respectively, based on the recommendation settings in BERT. The settings of the hyper-parameters related to the model architecture are identical to those of BERT_BASE_ [11]. Other hyper-parameters, such as the probabilities of *masked language model* and *next sentence prediction*, were set as the default values (15% and 50%, respectively). For either EhrBERT_500k_ or EhrBERT_1M_, we used four Tesla P40 graphics processing units to simultaneously fine-tune BioBERT on our EHR data. EhrBERT_500k_ takes approximately 12 hours per epoch and EhrBERT_1M_ takes approximately 23 hours per epoch.

### Models for Entity Normalization

**Figure 2:**
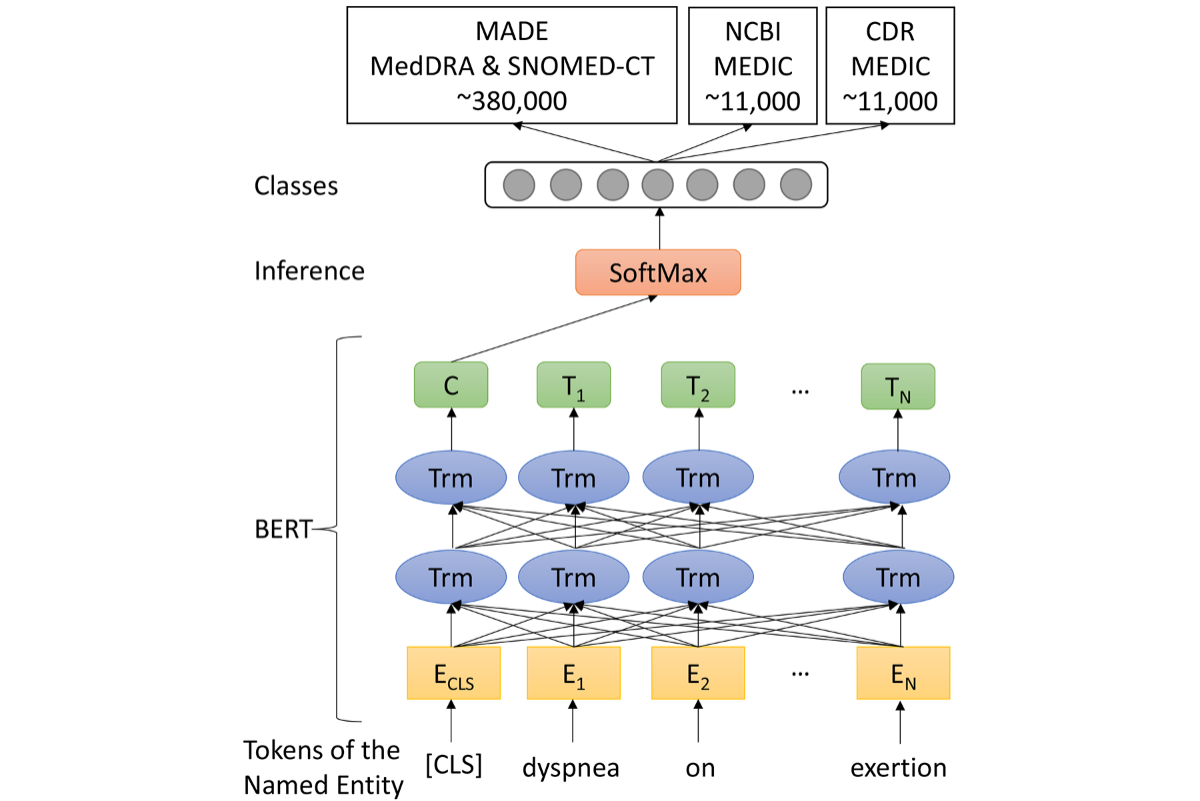
Model architectures. An example of entity normalization is shown and the named entity “dyspnea on exertion” is normalized to the term “60845006” in the Systematized Nomenclature of Medicine—Clinical Terms (SNOMED-CT) vocabulary (SNOMED International, 2019). The size of classes depends on the vocabularies used in a corpus, which is about 380,000 (Medical Dictionary for Regulatory Activities [MedDRA] and SNOMED-CT) for the Medication, Indication, and Adverse Drug Events (MADE) 1.0 corpus and 11,000 (MErged DIsease voCabulary [MEDIC]) for the National Center for Biotechnology Information (NCBI) Disease and Chemical-Disease Relations (CDR) corpora. BERT: bidirectional encoder representations from transformers; C: *d*^Trm^-dimensional representation; [CLS]: classifier token; E: *d*^emb^-dimensional embedding; T: *d*^Trm^-dimensional vector; Trm: bidirectional transformer.

As shown in Figure 2, we treated entity normalization as a text classification task. Following BERT and BioBERT, we employed the word representations from the top layer of transformers as the features for the normalization task. Concretely, a classifier token, [*CLS*], is padded before the given sequence of word pieces [28]. Thus, our model takes a sequence {[*CLS*], *w*_1_, ..., *w*_N_} as input. Here, *w*_n_ is not necessarily a word; it can also be a subword (aka, a word piece). Each word piece is mapped to a *d*^emb^-dimensional embedding, *E*_n_. In addition, the input also includes segment and position embeddings with the same dimension, *d*^emb^, which are mixed with the word piece embeddings.

After a few layers of bidirectional transformers, *Trm*, each word piece, *w*_n_, corresponds to a *d*^Trm^-dimensional vector, *T*_n_. The *d*^Trm^-dimensional representation, *C*, for the padding token, [*CLS*], is used as the representation of the whole sequence. Then *C* is input into the SoftMax layer to compute the probability distribution of all classes. The class with the maximal probability is selected as the prediction.

In terms of parameter initialization, the BERT part of the model was initialized with EhrBERT. Other parameters were randomly initialized with a uniform distribution. During training, the objective is to maximize the log-likelihood of gold annotations. We used the standard back-propagation to update all the parameters and the Adam algorithms [29] to control the update process. For hyper-parameter setting, *d*^emb^ and *d*^Trm^ are set as 768, the batch size is 32, the learning rate is 1e-5, and the dropout rate is 0.1. The training will stop early if the performance has not increased for 20 epochs.

### Corpora

We employed the Medication, Indication, and Adverse Drug Events (MADE) corpus [30], which derives from the MADE 1.0 challenge. The corpus includes 1089 EHR notes, which were divided into 876 notes for training and 213 notes for testing. This corpus contains the annotations of mapping adverse drug events to the Medical Dictionary for Regulatory Activities (MedDRA) [31] terms and of mapping indications, signs, and symptoms to the SNOMED-CT [32] terms. The MedDRA and SNOMED-CT vocabularies include about 380,000 terms in total, which are computed based on the MRCONSO.RRF file in the UMLS Metathesaurus, version 2016 AA. In the MADE corpus, there are about 35,000 and 8000 mentions in the training and test sets, respectively.

Moreover, we also employed two nonclinical corpora, namely the National Center for Biotechnology Information (NCBI) disease corpus [33] and the Chemical-Disease Relations (CDR) corpus [34], to evaluate EhrBERT in different domains. The NCBI disease corpus consists of 793 PubMed abstracts, 6892 disease mentions, and 790 unique disease concepts. The abstracts are split into 593, 100, and 100 for training, development, and testing, respectively. The CDR corpus is composed of 500, 500, and 500 PubMed abstracts for training, development, and testing, respectively. It includes 5818 disease mentions for normalization. The objectives of both corpora are to map each disease mention to a term in the MErged DIsease voCabulary (MEDIC) [35], which contains approximately 11,000 terms.

### Experimental Settings

For the MADE corpus, we utilized mention-level precision, recall, and F1 as evaluation metrics. A prediction is counted as *true-positive* only if both the boundary and term ID of the mention are correct. Besides using the gold entity mentions, we also utilized the mentions recognized by MetaMap [19] as the input for our models as a comparison. Because the outputs of MetaMap are the UMLS IDs [3], we also utilized the UMLS Metathesaurus to map SNOMED-CT and MedDRA terms to UMLS terms. During preprocessing, we transformed all the tokens in a mention or a term into lowercase. We also removed the punctuations but kept the numbers.

For the NCBI disease and CDR corpora, we utilized document-level precision, recall, and F1, following DNorm [1]. There are two ID sets for a document, namely the predicted ID set and the gold ID set. If a predicted ID is equal to a gold ID, we counted it as *true-positive*. The performance of the corpus is the macro-averaged performance of all documents. All the abbreviations are replaced with their full names using the dictionaries provided by DNorm. We employed gold mentions as input in order to compare with DNorm.

Besides precision, recall, and F1, we also analyzed statistical significances between different models. First, the MetaMap and DNorm were run once on test sets using their off-the-shelf models that were released by the authors. We believe that these models are elaborately tuned and can achieve the best performance as strong baselines. Second, the experiments for BERT, BioBERT, and EhrBERT were run thrice. During each run, we utilized a different random seed to initialize the model. After training, the model was run on the test set to obtain precision, recall, and F1. Lastly, the *t* test was utilized to determine if the performances of two models were statistically different based on the results of these runs.

## Results

Table 2 shows the F1s and the standard deviations of the models. The models are ranked from low to high based on F1s. Precisions and recalls are provided in Multimedia Appendix 1.

Tables 3-5 show the *P* values of the different models for the MADE (predicted entities), NCBI disease, and CDR corpora, respectively. The performance of the model along each row is lower than the performance of the model along each column, as shown in Table 2. We utilized .05 as the threshold to determine statistical significance.

The results for entity normalization are shown in Table 2. We ran our experiments thrice for all the models using different random seeds. The results in Table 2 are the mean F1 scores of these runs. We can see that no matter whether we used gold entities or MetaMap-predicted entities in the MADE corpus, EhrBERT performed better than BioBERT, and BioBERT performed better than BERT. In addition, BERT-based models obtained better results compared with MetaMap, improving the F1s by 2.22% for BERT, 2.28% for BioBERT, and 2.36% for both EhrBERT_500k_ and EhrBERT_1M_. From Tables 3-5, we can see that EhrBERT performed significantly better than MetaMap, BERT, and BioBERT. However, the performance differences between BERT, BioBERT, and EhrBERT are not always discernible.

In both the NCBI disease and CDR corpora, the F1s of BERT-based models were higher than the F1s of DNorm, as shown in Table 2. In the NCBI disease corpus, BioBERT achieved the highest F1 (90.71%). As shown in Tables 3-5, BioBERT is statistically discernible from BERT but not from EhrBERT. In the CDR corpus, BioBERT performed slightly worse than EhrBERT (93.42% vs 93.82%). In Tables 3-5, there are no statistical differences between BioBERT and EhrBERT_500k_, but a statistical difference exists between BioBERT and EhrBERT_1M_. The similar performances of EhrBERT and BioBERT may be because the domains of EhrBERT and BioBERT are close. Moreover, all models performed much better in the NCBI disease and CDR corpora than in the MADE corpus. One likely reason is that the class number of the MADE corpus is tens of times larger than those of the NCBI disease and CDR corpora.

Comparing EhrBERT_500k_ and EhrBERT_1M_, EhrBERT_1M_ consistently performed better in all the corpora as shown in Table 2. This implies that the size of the pretraining data may be a factor that influences the performance of BERT-based models. However, the significance analysis in Table 5 shows that the performance of EhrBERT_1M_ is only significantly different from that of EhrBERT_500k_ in the CDR corpus. There are no statistical differences between EhrBERT_500k_ and EhrBERT_1M_ in the other two corpora.

**Table 2:** F1s and standard deviations.

Corpus and model		F1 (%), mean (SD)	Improvement compared with MetaMap or DNorm^a^
**MADE^b^ (gold entities^c^)**			
	BERT^d^	67.87 (0.25)	N/A^e^
	BioBERT	68.22 (0.11)	N/A
	EhrBERT_500k_^f^	68.74 (0.14)	N/A
	EhrBERT_1M_^g^	68.82 (0.29)	N/A
**MADE (predicted entities^h^)**			
	MetaMap [19]	38.59 (0)	N/A
	BERT	40.81 (0.08)	+2.22
	BioBERT	40.87 (0.06)	+2.28
	EhrBERT_500k_	40.95 (0.04)	+2.36
	EhrBERT_1M_	40.95 (0.07)	+2.36
**NCBI^i^**			
	DNorm [1]	88.37 (0)	N/A
	BERT	89.43 (0.99)	+1.06
	EhrBERT_500k_	90.00 (0.48)	+1.63
	EhrBERT_1M_	90.35 (1.12)	+1.98
	BioBERT	90.71 (0.37)	+2.34
**CDR^j^**			
	DNorm [1]	89.92 (0)	N/A
	BERT	93.11 (0.54)	+3.19
	BioBERT	93.42 (0.10)	+3.50
	EhrBERT_500k_	93.45 (0.09)	+3.53
	EhrBERT_1M_	93.82 (0.15)	+3.90

^a^DNorm: disease name normalization.

^b^MADE: Medication, Indication, and Adverse Drug Events.

^c^We used gold entity mentions as input.

^d^BERT: bidirectional encoder representations from transformers.

^e^N/A: not applicable.

^f^EhrBERT_500k_: BERT-based model that was trained using 500,000 electronic health record notes.

^g^EhrBERT_1M_: BERT-based model that was trained using 1 million electronic health record notes.

^h^We used MetaMap-predicted entity mentions as input.

^i^NCBI: National Center for Biotechnology Information.

^j^CDR: Chemical-Disease Relations.

**Table 3:** *P* values of the different models for the Medication, Indication, and Adverse Drug Events (predicted entities) corpus.

Model	Model, *P* value			
	BERT^a^	BioBERT	EhrBERT_500k_^b^	EhrBERT_1M_^c^
MetaMap	<.001	<.001	<.001	<.001
BERT		.17	.02	.02
BioBERT			.04	.04
EhrBERT_500k_				.50

^a^BERT: bidirectional encoder representations from transformers.

^b^EhrBERT_500k_: BERT-based model that was trained using 500,000 electronic health record notes.

^c^EhrBERT_1M_: BERT-based model that was trained using 1 million electronic health record notes.

**Table 4:** *P* values of the different models for the National Center for Biotechnology Information disease corpus.

Model	Model, *P* value			
	BERT^a^	EhrBERT_500k_^b^	EhrBERT_1M_^c^	BioBERT
DNorm^d^	.10	.01	.04	.004
BERT		.25	.15	.03
EhrBERT_500k_			.37	.09
EhrBERT_1M_				.32

^a^BERT: bidirectional encoder representations from transformers.

^b^EhrBERT_500k_: BERT-based model that was trained using 500,000 electronic health record notes.

^c^EhrBERT_1M_: BERT-based model that was trained using 1 million electronic health record notes.

^d^DNorm: disease name normalization.

**Table 5:** *P* values of the different models for the Chemical-Disease Relations corpus.

Model	Model, *P* value			
	BERT^a^	BioBERT	EhrBERT_500k_^b^	EhrBERT_1M_^c^
DNorm^d^	.004	<.001	<.001	<.001
BERT		.18	.22	.04
BioBERT			.41	.03
EhrBERT_500k_				.03

^a^BERT: bidirectional encoder representations from transformers.

^b^EhrBERT_500k_: BERT-based model that was trained using 500,000 electronic health record notes.

^c^EhrBERT_1M_: BERT-based model that was trained using 1 million electronic health record notes.

^d^DNorm: disease name normalization.

## Discussion

### Principal Findings

As shown in the results, BERT-based models outperformed MetaMap and DNorm. However, the performance differences between BERT-based models are not quite as large. Therefore, any kind of BERT-based models should be effective for entity normalization if one does not pursue 1%-2% performance improvements. Moreover, we also found that the domain of pretrained data has an effect on BERT-based models, but the effect is slight by further adding pretrained data. We will discuss these topics in the following sections.

### Effect of Domains

In this section, we analyzed the effect of domains from two aspects. First, we investigated whether in-domain models performed better than out-domain models and whether the performance differences are statistically significant. For example, if the corpus belongs to the clinical domain (eg, MADE), the in-domain model (eg, EhrBERT) should theoretically perform better than out-domain models (eg, BERT or BioBERT). As shown in Multimedia Appendix 2 graph (a), in-domain models performed better than out-domain models in two corpora (ie, MADE and NCBI disease) out of three. In addition, statistical significance only emerged in the MADE corpus. By contrast, there are fewer corpora where out-domain models performed better than in-domain models. In the CDR corpus, the out-domain model (ie, EhrBERT) performed better than the in-domain model (ie, BioBERT); meanwhile, statistical significance exists. These results show that domains have an impact on the performances of models but the impact is not significantly visible between the biomedical and clinical domain.

Second, we analyzed whether clinical or biomedical domain models (eg, BioBERT or EhrBERT) performed better than general domain models (eg, BERT). As illustrated in Multimedia Appendix 2 graph (b), at least one model (ie, BioBERT or EhrBERT) of biomedical and clinical domains performed better than the general domain model (ie, BERT) in all corpora. More importantly, the performances of BioBERT or EhrBERT are significantly higher than that of BERT in all corpora. Therefore, the similarities of domains have a direct effect on the performances of models. Because biomedical and clinical domains are close to each other, the models trained using related data achieved similar results. By contrast, BERT achieved worse results in the biomedical or clinical corpora, since it was trained using the data from the general domain.

### Effect of the Data Size

In this section, we discuss the effect of the data size on the performance of EhrBERT. To this end, we split up our EHR notes for pretraining models into a smaller part (500,000 notes) (ie, EhrBERT_500k_) and a larger part (1 million notes) (ie, EhrBERT_1M_). From Table 2, we observed that EhrBERT_1M_ performed better than EhrBERT_500k_ in all corpora, improving the F1s by 0.08%, 0.35%, and 0.37%. Thus, it may be helpful to enlarge the size of pretraining data to generate high-quality models. However, the significance analysis in Tables 3-5 indicates that the performance of EhrBERT_1M_ is only statistically better than that of EhrBERT_500k_ in the CDR corpus. In other corpora, they are not statistically discernable. Therefore, we cannot reach the conclusion that the larger the size of the pretraining data, the better the model becomes. One likely reason is that EhrBERT was not pretrained from scratch. It was fine-tuned from BioBERT, which was fine-tuned from BERT. Thus, EhrBERT may only need a certain amount of data to transfer from one domain to another domain. For most downstream tasks, we believe that using EhrBERT_500k_ is effective enough. We leave further investigation of the data size for future work.

### Case Study

**Figure 3:**
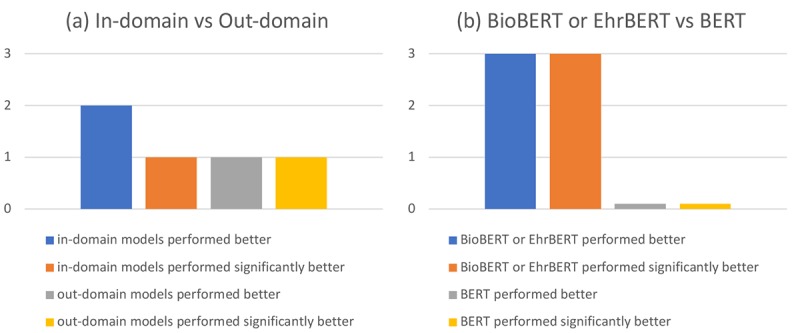
A case study. The left column shows examples where EhrBERT gave valid predictions. The right column shows examples where EhrBERT failed to give valid predictions. The rectangles denote mentions and weights of the word pieces in these mentions. The darker the color is, the larger the weight is. Split word pieces are denoted with “##.” The text in green and red indicate gold and predicted answers respectively. EhrBERT: bidirectional encoder representations from transformers (BERT)-based model that was trained using 1.5 million electronic health record notes.

To better understand EhrBERT, we manually analyzed about 100 cases in the MADE corpus and selected some typical cases that were predicted correctly or incorrectly. In addition, we also built a dot-attention [36] layer on top of EhrBERT to show the weight of each word. As illustrated in Figure 3, we learned the following points based on our observation.

First, short and simple entity mentions are easy to normalize. For example, the mention *fevers* was correctly normalized to the gold term *Fever* in the vocabulary. Moreover, complex words such as *osteoporosis* can be normalized correctly by our BERT-based models. In previous work, such words usually bring trouble, since they are out-of-vocabulary and cannot be well represented by models. However, our BERT-based models, which are built based on word pieces rather than words, can benefit from subword information and alleviate the out-of-vocabulary problem. Furthermore, long mentions, which consist of multiple words, are more difficult to be normalized. Through the visualization of attention weights, we found that EhrBERT can sometimes make valid predictions by ﻿concentrating on keywords and by neglecting noise at the same time. For instance, since our model paid more attention to *weight* and *gain* in the mention *weight loss or gain*, it successfully linked the mention to the correct term, *Weight gain*.

Through the case study, we also learned some lessons. First, EhrBERT sometimes paid more attention to irrelevant words, leading to incorrect predictions. For example, since EhrBERT gave more attention to *calculus* in the mention *ureteral calculus*, it missed the important information from *ureteral*. Therefore, it linked the mention *ureteral calculus* to an invalid term, *Kidney stone*. Second, as the mention lengths became longer, it was more difficult for EhrBERT to focus on the correct words. For example, regarding the mention *complications of his stone retrieval*, since EhrBERT concentrated on the part near *stone* rather than *complications*, it linked the mention to *Kidney stone* rather than to the valid term *Complication of procedure*. Third, we found that even though EhrBERT sometimes paid more attention to proper words, it still failed to make correct predictions. For example, *body* and *ache* attained higher weights in the mention *body aches*, but the mention was not linked to the right term, *Pain*. One likely reason is that the model needs to truly understand the similarity between *Pain* and *ache*. Lastly, we observed some cases that are difficult even for us. For instance, the mention *reactions to drugs* is ambiguous. It is hard to know the true reason for *reaction* based on limited information. Therefore, such a situation may need more information to disambiguate mentions, such as context or background knowledge.

### Limitations

One limitation of our work is that entity normalization is treated as a single-label classification problem; however, it is not possible to handle this type of problem when an entity can be linked to more than one term in the vocabulary. To address this limitation, one could leverage ﻿the multi-label classification approach [37] via the binary cross-entropy loss to train the model. Another limitation is that our model has not made full use of the information in vocabularies, such as synonyms and hierarchical relationships. In the future, this can be explored via other models such as graph convolutional neural networks [38]. Lastly, we have observed that there is a bias in our model as shown in Multimedia Appendix 3. Like most machine learning models, our model prefers highly frequent words in the dataset.

### Conclusions

In this paper, we investigated the effectiveness of BERT-based models for the entity normalization task in the biomedical and clinical domain. We found that BERT-based normalization models outperformed some state-of-the-art systems. Moreover, the performance can be further improved by pretraining our models on large-scale EHR notes. Furthermore, we found that domains have an impact on the performance of BERT-based models. The impact depends on the similarities between the domains of models and tasks. In the future, our approach will be evaluated in more clinical NLP tasks.

## Appendix

Multimedia Appendix 1 Full results on three corpora of entity normalization.

Multimedia Appendix 2 Statistics of the domain effect.

Multimedia Appendix 3 Analysis of the model bias.

## Abbreviations

BERT: bidirectional encoder representations from transformers
C: *d*^Trm^-dimensional representation
CDR: Chemical-Disease Relations
[*CLS*]: classifier token
DNorm: disease name normalization
E: *d*^emb^-dimensional embedding
EHR: electronic health record
EhrBERT: BERT-based model that was trained using 1.5 million electronic health record notes
EhrBERT_1M_: BERT-based model that was trained using 1 million EHR notes
EhrBERT_500k_: BERT-based model that was trained using 500,000 EHR notes
ELMo: embeddings from language models
EN: entity normalization
MADE: Medication, Indication, and Adverse Drug Events
MedDRA: Medical Dictionary for Regulatory Activities
MEDIC: MErged DIsease voCabulary
NCBI: National Center for Biotechnology Information
NLP: natural language processing
PMC: PubMed Central
SNOMED-CT: Systematized Nomenclature of Medicine—Clinical Terms
T: *d*^Trm^-dimensional vector
Trm: bidirectional transformer
UMLS: Unified Medical Language System
*w*: word piece
