# Supplementary material for: Fine-Tuning Bidirectional Encoder Representations From Transformers (BERT)–Based Models on Large-Scale Electronic Health Record Notes: An Empirical Study
Source: JMIR Med Inform. 2019 Sep 12;7(3):e14830. doi: 10.2196/14830 (PMC6746103; doi:10.2196/14830)
Supplement: Multimedia Appendix 1 [file medinform_v7i3e14830_app1.pdf]

Appendix 1: Full Results on 3 Corpora of Entity Normalization

Table 1. Results of entity normalization. Models are ranked from low to high based on F1s. "gold entities" and "predicted entities" mean we used gold entity mentions and MetaMap-predicted entity mentions as input.

Corpus	Model	Precision	Recall	F1
MADE (gold entities)	BERT	67.87	67.87	67.87
	BioBERT	68.22	68.22	68.22
	$EhrBERT_{500K}$	68.74	68.74	68.74
	$EhrBERT_{1M}$	68.82	68.82	68.82
MADE (predicted entities)	MetaMap [1]	34.24	44.22	38.59
	BERT	36.16	46.84	40.81
	BioBERT	36.21	46.90	40.87
	$EhrBERT_{500K}$	36.28	46.99	40.95
	$EhrBERT_{1M}$	36.28	46.99	40.95
NCBI	DNorm [2]	90.67	88.67	88.37
	BERT	88.63	91.23	89.43
	$EhrBERT_{500K}$	89.30	91.69	90.00
	$EhrBERT_{1M}$	89.77	91.90	90.35
	BioBERT	90.07	92.27	90.71
CDR	DNorm [2]	91.34	89.71	89.92
	BERT	92.59	94.08	93.11
	BioBERT	93.06	94.21	93.42
	$EhrBERT_{500K}$	93.06	94.26	93.45
	$EhrBERT_{1M}$	93.44	94.64	93.82

References

1. Aronson AR, Lang F-M. An overview of MetaMap: historical perspective and recent advances. J Am Med Inform Assoc 2010;17(3):229–236.

2. Leaman R, Islamaj Dogan R, Lu Z. DNorm: disease name normalization with pairwise learning to rank. Bioinforma Oxf Engl 2013 Nov 15;29(22):2909–2917. PMID:23969135
