# Supplementary material for: Fine-Tuning Bidirectional Encoder Representations From Transformers (BERT)–Based Models on Large-Scale Electronic Health Record Notes: An Empirical Study
Source: JMIR Med Inform. 2019 Sep 12;7(3):e14830. doi: 10.2196/14830 (PMC6746103; doi:10.2196/14830)

Appendix 2: Statistics of the domain effect

Figure 1. Statistics of the domain effect. (a) shows the counts that in-domain or out-domain models performed better in 3 corpora. In-domain models indicate EhrBERT for the MADE corpus, BioBERT for the NCBI and CDR corpora. (b) shows the counts that biomedical-clinical-domain models (BioBERT or EhrBERT) and general-domain models (BERT) performed better in 3 corpora.

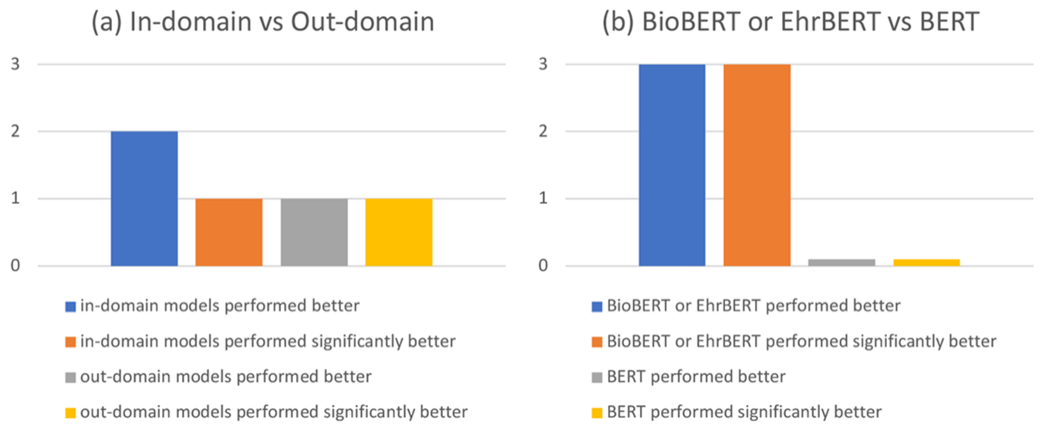

Supplement: Multimedia Appendix 2 [file medinform_v7i3e14830_app2.pdf]
