# Supplementary material for: Fine-Tuning Bidirectional Encoder Representations From Transformers (BERT)–Based Models on Large-Scale Electronic Health Record Notes: An Empirical Study
Source: JMIR Med Inform. 2019 Sep 12;7(3):e14830. doi: 10.2196/14830 (PMC6746103; doi:10.2196/14830)
Supplement: Multimedia Appendix 3 [file medinform_v7i3e14830_app3.pdf]

Appendix 3: Analysis of the Model Bias

Figure 1. Word frequencies for correctly- and incorrectly-predicted entities.

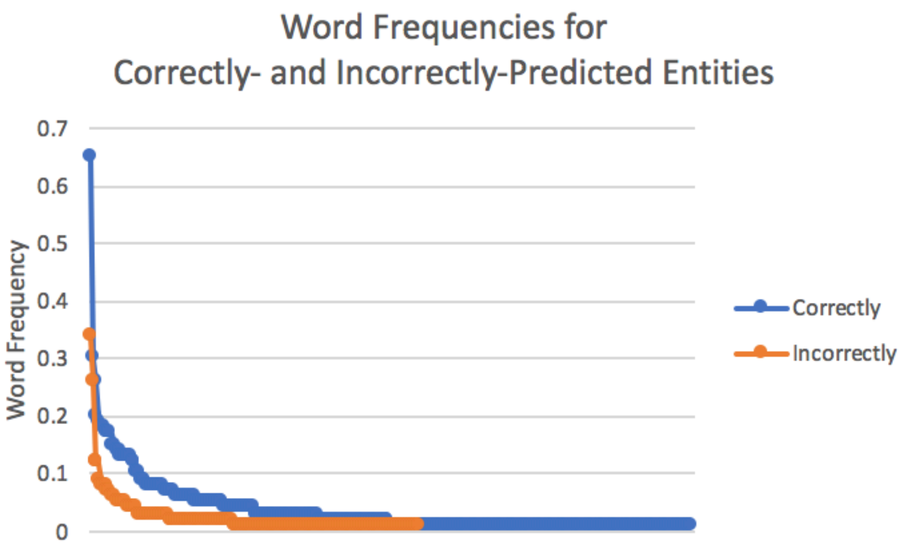

To analyze the bias of our model, we conducted the following experiment. First, we divided the predicted results of EhrBERT on the test set of the MADE corpus into two categories, correctly-predicted entities (i.e., entities are normalized into correct terms) and incorrectly-predicted entities. Second, we counted the frequency of each word occurred in the entities of each category, respectively. The statistical results are illustrated in Figure 1. We can see that the frequencies of the words in incorrectly-predicted entities are lower than those in correctly-predicted entities. This demonstrates our model performs better for the normalization of high-frequent words. One likely reason is that our model is not able to learn efficient representations for low-frequent words due to their inadequate training. Like most machine learning models, our model also suffers from the bias of the training data.
